# Efficacy of anlotinib in Chinese patients with metastatic breast cancer: A retrospective observational study

**DOI:** 10.1111/jcmm.18008

**Published:** 2023-10-27

**Authors:** Shuochuan Liu, Wenxiang Zhi, Lu Zhang

**Affiliations:** ^1^ Department of Breast disease, Henan Breast Cancer Center The Affiliated Cancer Hospital of Zhengzhou University & Henan Cancer Hospital Zhengzhou Henan Province China; ^2^ Deprtment of Ultrasonography, Fudan University Shanghai Cancer Center, Department of Oncology, Shanghai Medical College Fudan University Shanghai China; ^3^ Department of Combine Traditional Chinese & Western The Affiliated Cancer Hospital of Zhengzhou University & Henan Cancer Hospital Zhengzhou Henan Province China

**Keywords:** anlotinib, efficacy, metastatic breast cancer, safety, tyrosine kinase inhibitor

## Abstract

Anlotinib, a multitarget tyrosine kinase inhibitor, can inhibit tumour angiogenesis proliferation, metastasis, promote vascular normalization, increase T cell and NK cell activity and infiltration, remodel tumour microenvironment and synergistic immune enhancement. *Our study aimes* to evaluate the efficacy of anlotinib in the treatment of advanced metastatic breast cancer (MBC) after multiple lines of therapy. Patients *included were* treated with anlotinib for advanced MBC in the Affiliated Cancer Hospital of Zhengzhou University from 1 January 2019 to 30 June *2023.* The objective remission rate, disease‐free *progression* survival and adverse reactions were analysed. We compared and analysed the efficacy of anlotinib in the treatment of advanced metastatic breast cancer, which showed that ORR was 23.6% and DCR was 69.1%. The DCR of monotherapy was 66.7% and that of combination therapy was 69.6% in MBC patients. The combination therapy, combined with chemotherapy had the best effect (79.3%), combined with immunotherapy came second. In addition, the DCR (88.9%) was higher in MBC patients having received prior antiangiogenic therapy. According to the Kaplan–Meier (K‐M) survival estimate analysis, the mPFS was 4.17 months (95% CI, 1.758–6.582 months) in Her‐2 positive MBC patients, and 7.83 months (95% CI, 2.416–9.104) in Her‐2 negative MBC patients. The mPFS was 5.76 months (95% CI, 3.231–8.298 m) in HR positive MBC patients, 7.83 months (95% CI, 3.182–12.478 m) in TNBC patients. Fatigue (20.0%), hypertension (21.8%) and liver dysfunction (18.2%) were common adverse reactions, followed by bone marrow suppression (16.4%), anorexia (14.5%), hypothyroidism (14.5%) and diarrhoea (14.5%). Altogether, Anlotinib monotherapy or combination therapy provides a viable third (or above)‐line therapeutic strategy in patients with metastatic breast cancer. The adverse reactions of anlotinib are well tolerated and controllable.

## 1 INTRODUCTION

Breast cancer, a malignant tumour in the glandular epithelium of the breast, occurs in either women (making up 99%) or men (1%),^1^, ^2^ and can metastasize to important organs such as bone, lung, liver and brain. According to the International Agency for Research on Cancer (IARC) of the World Health Organization (2020), the number of new cases of breast cancer reached 2.26 million, surpassing that (2.2 million) of lung cancer for the first time.^3^ In China, the National Cancer Center has reported that breast cancer has become the most prevalent cancer in women.^4^ The two epidemiological characteristics of breast cancer in China include: early age, late clinical stage at diagnosis. For patients with Her‐2 positive advanced breast cancer, a PHOEBE study showed median progression‐free survival was significantly longer with pyrotinib‐combined regiments (12.5 months [95% CI 9.7‐not reached]) than with lapatinib plus capecitabine (6.8 months [95% CI 5.4‐8.1]).^5^ The SOPHIA China research data indicate that Margetuximab is effective and well tolerated in the Chinese population, which is consistent with its efficacy in the global population.^6^ In addition, a retrospective study in France, which used Bevacizumab as the first‐line treatment for Her‐2 negative breast cancer patients, also demonstrated the advantages in controlling tumour angiogenesis.^7^, ^8^ Related guidelines have been modified based on recent clinical research data, which offers us inspirations in the treatment of advanced breast cancer.

To hinder tumour angiogenesis, anlotinib acts on various pathways involving Vascular endothelial growth factor (VEGFR) 1, VEGFR3, VEGFR2/kinase insert domain receptor (KDR), platelet‐derived growth factor receptor‐a (PDGFR‐a), c‐Kit or fibroblast growth factor receptor (FGFR). Anlotinib has a lower IC50 for VEGFR, PDGFR and FGFR, which can inhibit cell proliferation and metastasis.^9^, ^10^, ^11^ In addition, anlotinib combined with chemotherapy/TKI can significantly improve the tumour response rate. Therefore, we conducted a real‐world retrospective study on the evaluation of the efficacy of anlotinib in the treatment of advanced metastatic breast cancer.

### 1.1 Data source and study population

This study was a retrospective single‐center analysis conducted in the Affiliated Cancer Hospital of Zhengzhou University. Metastatic breast cancer patients with the failure of standard treatment and then receiving anlotinib‐based treatment at our hospital as the second (or above)‐line treatment between December 2019 and June 2023 were enrolled (201910226). We collected the medical data of 55 female patients. These patients aged 32–80 years with Eastern Cooperative Oncology Group (ECOG) performance status of 0–2.

### 1.2 Treatment methods

Patients were treated with anlotinib in combination with chemotherapy, immunotherapy, anti‐Her‐2 drugs or hormonotherapy. Chemotherapy drugs mainly included gemcitabine, capecitabine, vinorelbine, albumin‐bound paclitaxel, docetaxel and etoposide capsules. Gemcitabine (1000 mg/m^2^) and vinorelbine (25 mg/m^2^) were given at days 1 and 8; capecitabine (1000 mg/m^2^) was given twice daily for 14 days; a combination of Albumin‐bound paclitaxel (175 mg/m^2^) and docetaxel (75 mg/m^2^) was given at day 1; these prescriptions made up a course of 3 weeks. Etoposide capsules were prescribed as 175 mg daily for 5 consecutive days and then discontinued for 3 weeks. Tislelizumab (200 mg every 3 weeks) or toripalimab (3 mg/kg every 2 weeks), an antihuman programmed cell death 1 (PD‐1) monoclonal antibody for immunotherapy, was also administered. Her‐2‐targeting therapies were also used in Her‐2 positive breast cancer patients. ApatinibMesylate Tablets (850 mg) were given once a day until it occurs an unacceptable adverse reaction or progression. Physicians determined a dosage based on patients' tolerance to treatment and any adverse reactions during the process, as well as their height, weight and disease severity. Anlotinib tablets in three sizes (8 mg, 10 mg and 12 mg) were taken into an empty stomach in the morning for continuous 2 weeks and then discontinued for 1 week.

### 1.3 Clinical efficacy

Having taken anlotinib tablets for two consecutive weeks, the patient was rechecked with enhanced CT or MRI for the short‐term efficacy of Response Evaluation Criteria in Solid Tumours (RECIST)1.1. Complete Remission (CR): all target lesions disappeared completely. All pathological lymph nodes must be reduced to normal size, partial response (PR): the sum of the diameters of all measurable target lesions is reduced by ≥30%, progressive disease (PD): the minimum value of the sum of the diameters of all measured target lesions is increased by 20% or any new lesion appears, disease stability (SD): refers to conditions between PD and PR. The primary end point was progression‐free survival (PFS), secondary end points included objective response rate (ORR), disease control rate (DCR) and safety. ORR was defined as the proportion of patients who presented a complete response (CR) or a partial response (PR) to therapy. DCR was defined as the proportion of patients who achieved CR, PR and stable disease (SD) in response to therapy. PFS was estimated using the Kaplan–Meier method.

### 1.4 Statistical analysis

Qualitative variables were expressed as counts and percentages, and compared using Pearson's chi‐squared or Fisher tests, while quantitative variables as mean and standard deviation or median and compared by Student's test. Patients who received ≥2 courses of anlotinib were included into survival and safety analyses. The Kaplan–Meier method was used to estimate PFS. ORR and DCR comparisons were performed with Fisher's exact test. All analyses were performed separately in patients with Her‐2+, HR+/Her‐2‐and triple‐negative breast cancer (TNBC). Data were processed using SPSS version 22.0.

## 2 RESULTS

### 2.1 Baseline characteristics

A total of 55 patients with MBC who received anlotinib as second (or above)‐line therapy were included. Patients and treatment characteristics are summarized in Table 1. The median age was 55 years (range 32–80 years). Ten (18.2%) patients were Her‐2 positive, while the others (81.8%) were hormone receptor positive/Her2‐negative (40.0%) or triple‐negative (41.8%). Most patients (51,92.7%) were ECOG PS 1–2. The median number of treatment lines was five, and most patients (42,76.4%) had already received more than four treatment lines before the use of anlotinib. The metastasis was divided into visceral (41, 74.5%) and nonvisceral (14, 25.5%). In addition, the most common sites of metastasis included liver (27.3%), lung (60.0%), bone (38.2%) and brain (10.9%) and lymph node (96.4%). Anlotinib was used in a monotherapy (16.4%), or combined with chemotherapy (52.7%), immunotherapy (18.2%), Her‐2 drugs (9.1%) and endocrine drugs (3.6%).

**TABLE 1:** Baseline characteristics of 55 MBC patients.

Characteristics	Patients (*n* = 55)
Age, years, median (range)	55 (32–80)
Molecular subtyping	
Her‐2+	10 (18.2%)
Her‐2‐	45 (81.8%)
HR+	22 (40.0%)
TNBC	23 (41.8%)
Treatment line, median (range)	5 (2–14)
2	5 (9.1%)
3	8 (14.5%)
≥4	42 (76.4%)
ECOG PS	
0	4 (7.3%)
1	19 (34.5%)
2	32 (58.2%)
Metastasis type	
Visceral	41 (74.5%)
Nonvisceral	14 (25.5%)
Metastatic site	
Liver	15 (27.3%)
Lung	33 (60.0%)
Brain	6 (10.9%)
Bone	21 (38.2%)
Lymph nodes	53 (96.4%)
Number of metastatic sites, n (%)	
1	4 (7.3%)
≥2	51 (92.7%)
Prior antiangiogenic therapy	
Yes	9 (16.4%)
No	46 (83.6%)
Treatment regimens	
Monotherapy	9 (16.4%)
Combined with chemotherapy	29 (52.7%)
Combined with immunotherapy	10 (18.2%)
Combined with Her‐2 drugs	5 (9.1%)
Combined with hormonotherapy	2 (3.6%)

Abbreviations: ECOG, Eastern Cooperative Oncology Group; HR, hormone receptor; MBC, metastatic breast cancer; TNBC, triple‐negative breast cancer.

### 2.2 Efficacy

We analysed the efficacy of anlotinib in MBC patients stratified according to molecular subtype, treatment regimen and prior antiangiogenic therapy (Table 2). In all the patients, the ORR was 23.6% and the DCR was 69.1%. Both ORR and DCR were significantly lower in Her‐2 positive patients than in Her‐2 negative patients. The DCR in the monotherapy group was not different from those in combination therapy groups, but ORR was higher in the monotherapy group, which might be related to the earlier use of anlotinib (e.g. in the second or third line). In the combination therapy groups, both ORR and DCR were significantly superior to those in other regimens. In addition, we found that DCR was higher in the patients receiving prior antiangiogenic therapy.

**TABLE 2:** The efficacy of anlotinib treatment in patients with metastatic breast cancer.

Parameter	Short‐term efficacy				ORR	DCR
	CR	PR	SD	PD		
Total	1	12	25	17	23.6%	69.1%
Molecular subtyping						
Her‐2+	0	1	4	5	10.0%	50.0%
Her‐2‐	1	11	21	12	26.7%	73.3%
HR+	1	7	8	6	36.4%	72.7%
TNBC	0	4	13	6	17.4%	73.9%
Treatment type						
Monotherapy	0	5	1	3	55.6%	66.7%
Combination	1	7	24	14	17.4%	69.6%
Combination type						
Combined with chemotherapy	1	6	16	6	24.1%	79.3%
Combined with immunotherapy	0	1	6	3	10.0%	70.0%
Combined with Her‐2 drugs	0	0	2	3	0	40.0%
Combined with hormonotherapy	0	0	0	2	0	0
Prior antiangiogenic therapy						
Yes	0	2	6	1	22.2%	88.9%
No	1	10	19	16	23.9%	65.2%

Abbreviations: CR, complete response; DCR, disease control rate; ORR, objective response rate; PD, progressive disease; PR, partial response; SD, stable disease.

*Note*: Short‐term efficacy was classified by modified Response Evaluation Criteria in Solid Tumours version 1.1 (RECIST 1.1).

### 2.3 Survival

#### 2.3.1 Survival in MBC patients of different molecular subtypes

**FIGURE 1:**
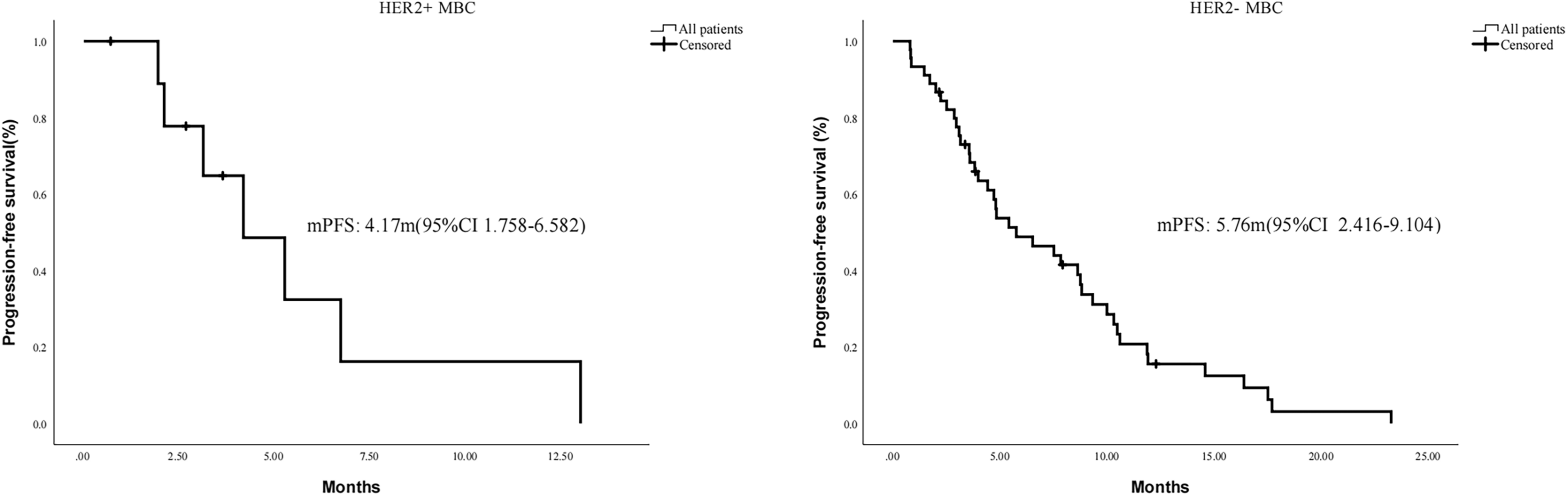
PFS curve plots in MBC patients either Her‐2 negative or Her‐2 positive.

According to the K‐M survival analysis (Figure 1), the median PFS of MBC patients with Her‐2 positive was 4.17 months (95% CI, 1.758–6.582 m), and shorter than that in MBC patients with Her‐2 negative (95% CI, 2.416–9.104 m).

#### 2.3.2 Survival in Her‐2 negative MBC patients

**FIGURE 2:**
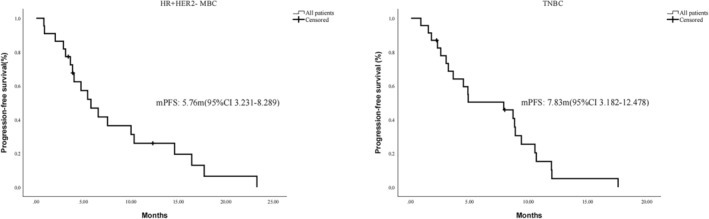
PFS in MBC patients either HR negative or HR positive. mPFS, median progression‐free survival; MBC, metastatic breast cancer; HR, hormone receptor; TNBC, triple‐negative breast cancer; CI, confidence interval.

Her‐2 negative MBC was classified into include TNBC and HR positive breast cancer. According to K‐M curve (Figure 2), the mPFS was 5.76 months (95% CI, 3.231–8.298 m) in HR positive MBC patients and 7.83 months (95% CI, 3.182–12.478 m) in TNBC patients, with a difference of nearly 2 months which suggested that the greater short‐term benefit of anlotinib for triple‐negative breast cancer.

#### 2.3.3 Adverse events

No fatal adverse reactions were found during the study period (Table 3). Most adverse reactions were tolerated, and the patients could continue to take anlotinib after drug intervention or adjustment of dosage. Fatigue (20.0%), hypertension (21.8%) and liver dysfunction (18.2%) were common adverse reactions. Due to serious fatigue, the dose was adjusted in one patient. Four patients (≥ grade2) were given antihypertensive drugs to treat hypertension. Three patients (≥grade2) adjusted the dose because of hand‐foot syndrome (12.7%). Anorexia (14.5%), bone marrow suppression (16.4%), hypothyroidism (14.5%) and diarrhoea (14.5%) were also reported (Table 3). The adverse reactions were classified according to the standard of common drug toxic reactions of National Cancer Institute (Version 4.0).

**TABLE 3:** Treatment‐related adverse events.

Adverse events	All grades, *n* (%)	Grade 1, *n* (%)	Grade 2, *n* (%)	Grades 3–4, *n* (%)
Fatigue	11 (20.0)	8 (14.5)	2 (3.6)	1 (1.8)
Anorexia	8 (14.5)	5 (9.1)	3 (5.5)	0 (0)
Weight loss	5 (9.1)	3 (5.5)	2 (3.6)	0 (0)
Bone marrow suppression	9 (16.4)	6 (10.9)	3 (5.5)	0 (0)
Hypertension	12 (21.8)	8 (14.5	3 (5.5)	1 (1.8)
Mucositis oral	8 (14.5)	6 (10.9)	2 (3.6)	0 (0)
Hand‐foot syndrome	7 (12.7)	4 (7.3)	2 (3.6)	1 (1.8)
Hypothyroidism	8 (14.5)	5 (9.1)	3 (5.5)	0 (0)
Haemoptysis	6 (10.9)	4 (7.3)	2 (3.6)	0 (0)
Hyperlipemia	8 (14.5)	5 (9.1)	3 (5.5)	0 (0)
Abdominalgia	4 (7.3)	3 (5.5)	1 (1.8)	0 (0)
Diarrhoea	8 (14.5)	5 (9.1)	3 (5.5)	0 (0)
Liver dysfunction	10 (18.2)	9 (16.4)	1 (1.8)	0 (0)
Proteinuria	5 (9.1)	3 (5.5)	2 (3.6)	0 (0)
Anaemia	4 (7.3)	3 (5.5)	1 (1.8)	0 (0)

## 3 DISCUSSION

Emerging treatments have improved the survival rate of patients with breast cancer, but metastasis and recurrence remain huge clinical challenges.^12^, ^13^ For those with MBC, the treatment has shifted its focus to reducing adverse reactions, improving the quality of life and prolonging the survival.

Perou et al. have subdivided breast cancer into four main clinical subtypes, according to gene expression profiles and receptor status (oestrogen receptor [ER], progestogen receptor [PR] and human epidermal growth factor receptor 2 [Her‐2]), as well as proliferation status^10^ assessed by Ki67.^14^ Clinical subtypes (in the order of invasiveness) include lumen A, lumen B, Her‐2 overexpression and basal/triple negative. Each subtype demonstrates organ‐specific metastasis. MBC is more likely to metastasize to liver (15%–32.0%), lung (21%–32.0%), bone (30%–60.0%) and brain (4%–10.0%).^15^, ^16^ The proportion of brain metastasis is relatively low, which may be related to a special brain structure (blood–brain barrier).^17^ A total of 55 patients were included in this study, 15 patients with liver metastasis (27.3%), 33 patients with lung metastasis (60.0%), six patients with brain metastasis (10.9%) and 21 patients with bone metastasis (38.2%), which is consistent with previous studies.^18^, ^19^, ^20^, ^21^ It is one of the most common ways for breast cancer cells to metastasize to regional lymph nodes through lymphatic vessels, and our research showed that more than 95.0% of patients had lymph node metastasis. Moreover, cancer cells can also metastasize to organs throughout the body by bloodstream.

‐Endocrine drugs combined with Cyclin‐dependent kinases 4 and 6 (CDK4/6) inhibitors are regarded as the standard first‐line treatment plan for patients without visceral crisis. For the third (or above)‐line treatment, tukatinib in combination with trastuzumab (no local intervention required), lapatinib and neratinib (no or stable metastatic breast cancer) in combination with capecitabine are recommended for Her‐2 positive patients.^22^, ^23^ Unfortunately, lapatinib was taken off from the Health Care Directory in 2019 for a variety of reasons, Domestic Pyrotinib Maleate Tablets was included in the same year, while these drugs are expensive and insufficiently supplied, making their use highly limited.^24^ Clinical trials have confirmed that the first‐line combination of antiangiogenesis drugs (bevacizumab) and chemotherapy can improve the progression‐free survival rate of advanced metastatic breast cancer.^25^ Multitarget small molecule inhibitors, such as anlotinib, have been produced in China and shown potential advantages.^10^, ^26^

In terms of overall treatment efficacy, the ORR of single therapy with anlotinib is significantly better than that of the combined regimen,^27^ while the DCR of the them shows opposite results. Compared with Miyashita M's study, the combination treatment group had significantly improved PFS.^28^ It should be noted that in this study only the best efficacy was evaluated. We conducted research on five patients (PR). In one patient (SD) who received single therapy with anlotinib, we found that the remission time and treatment course were shorter with single use of anlotinib, meanwhile, in the one patient (CR), seven patients (PR), 24 patients (SD) receiving combined regimens, the DCR of patients in the combined regimen reached about 70.0%, and the DCR of patients in the combined chemotherapy reached 79.3%. However, due to adverse drug reactions, patients in the combined chemotherapy had to extend the treatment interval.

Although the preliminary results of the 2022 ESMO conference report show that patients with advanced TNBC have a higher tumour burden and more gene mutations, treatments can be based on corresponding targets.^23^ The treatment strategy is stratified based on programmed cell death 1 ligand 1(PD‐L1) status and gBRCA mutation status, recommend SG as 2 L treatment for TNBC patients,^29^ if it is a patient with low Her‐2 expression, T‐DXd is recommended as a choice for first‐line chemotherapy progression.^30^

Previous studies have shown that low‐dose antitumour angiogenic drugs can promote vascular normalization,^31^, ^32^ improved the tumour microenvironment and sensitized radiotherapy,^33^ Other studies have shown that anlotinib can increase T cell activity and infiltration by downregulating the expression of PD‐L1 in endothelial cells,^34^ which can also reshape the tumour microenvironment by increasing the infiltration of innate immune cells (Natural killer cell, T cell and B cell).^35^ The immunotherapy of Nivolumab and Yipimumab has shown initial efficacy in the new adjuvant treatment.^36^ However, considering the low objective response rate of 5.0%–23%, the single use of immunosuppressive drugs is not recommended.^37^ The treatment options may vary with molecular types of MBC.

In this study, patients with metastatic breast cancer after multiline treatment were included, and few options were available for them. This study showed that the DCR of anlotinib combined with chemotherapy and immunotherapy could reach 79.3% and 70.0%, which is superior to the combination of Her‐2 drugs and hormone therapy. And the median survival period for Her‐2+ individuals was 4.17 months, while that for Her‐2‐ individuals reached 5.76 months, which is similar to the results of Hu et al.^38^ We further conducted subgroup analysis of Her‐2‐patients, and found that when HR was positive, the median survival was 5.76 months. In TNBC patients, the median survival was 7.83 months. We found that the median survival of Her‐2‐patients was better than that of Her‐2 + patients, which is similar to the results of Yu et al.^39^ This difference may be related to the early use of anlotinib. Patients with Her‐2 + can be associated with the use of anti‐Her‐2 drugs or new antibody‐drug conjugate (ADC) drugs, resulting in later use of anlotinib.

In addition, we observed that early use of antitumour angiogenesis drugs also increased the DCR of MBC. This also provides a new direction for us to treat advanced metastatic breast cancer. We have to acknowledge that this study is limited by a small sample size and lacks statistical analysis. However, no fatal adverse reactions of anlotinib were found in this retrospective study. The dosage is also controllable. Nevertheless, the evidence in this study may provide a prospect of using anlotinib for advanced metastatic breast cancer.

## 4 CONCLUSION

Anlotinib monotherapy or combination therapy provides a viable third (or above)‐line therapeutic strategy in patients with metastatic breast cancer. The adverse reactions of anlotinib are well tolerated and controllable.

## AUTHOR CONTRIBUTIONS

**Shuochuan Liu:** Writing – original draft (lead). **Wenxiang Zhi:** Writing – review and editing (equal). **lu zhang:** Writing – review and editing (equal).

## FUNDING INFORMATION

The authors' current work on metastasis breast cancer is funded by Project of Henan Provincial Health Commission (2022ZY1223) and Natural Science Foundation of Shanghai (23ZR1412400).

## CONFLICT OF INTEREST STATEMENT

The authors report no conflicts of interest in this work.

## Data Availability

Research data are not shared.
